# The *Drosophila* Retinoblastoma Binding Protein 6 Family Member Has Two Isoforms and Is Potentially Involved in Embryonic Patterning

**DOI:** 10.3390/ijms160510242

**Published:** 2015-05-06

**Authors:** Rodney Hull, Brent Oosthuysen, Umar-Faruq Cajee, Lehlogonolo Mokgohloa, Ekene Nweke, Ricardo Jorge Antunes, Theresa H. T. Coetzer, Monde Ntwasa

**Affiliations:** 1 School of Molecular & Cell Biology, University of the Witwatersrand, Johannesburg, South Africa Private Bag 3, WITS-2050 Johannesburg, South Africa; E-Mails: hullr@unisa.ac.za (R.H.); brent.oosthuysen@gmail.com (B.O.); Umar.Cajee@students.wits.ac.za (U.-F.C.); hlogzas@gmail.com (L.M.); Ekene.Nweke@students.wits.ac.za (E.N.); rickyant@gmail.com (R.J.A.); 2 School of Life Sciences, University of KwaZulu-Natal (Pietermaritzburg campus); 3209 Scottsville, South Africa; E-Mail: Coetzer@ukzn.ac.za

**Keywords:** retinoblastoma binding protein 6, RBBP6, SNAMA, p53, Rb, Mdm2

## Abstract

The human retinoblastoma binding protein 6 (RBBP6) is implicated in esophageal, lung, hepatocellular and colon cancers. Furthermore, RBBP6 was identified as a strong marker for colon cancer prognosis and as a predisposing factor in familial myeloproliferative neoplasms. Functionally, the mammalian protein interacts with p53 and enhances the activity of Mdm2, the prototypical negative regulator of p53. However, since RBBP6 (known as PACT in mice) exists in multiple isoforms and *pact^−/−^* mice exhibit a more severe phenotype than *mdm2^−/−^* mutants, it must possess some Mdm2-independent functions. The function of the invertebrate homologue is poorly understood. This is complicated by the absence of the *Mdm2* gene in both *Drosophila* and *Caenorhabditis elegans*. We have experimentally identified the promoter region of *Snama*, the *Drosophila* homologue, analyzed potential transcription factor binding sites and confirmed the existence of an additional isoform. Using band shift and co-immunoprecipitation assays combined with mass spectrometry, we found evidence that this gene may be regulated by, amongst others, DREF, which regulates hundreds of genes related to cell proliferation. The potential transcription factors for *Snama* fall into distinct functional groups, including anteroposterior embryonic patterning and nucleic acid metabolism. Significantly, previous work in mice shows that *pact^−/−^* induces an anteroposterior phenotype in embryos when rescued by simultaneous deletion of p53. Taken together, these observations indicate the significance of RBBP6 proteins in carcinogenesis and in developmental defects.

## 1. Introduction

The human retinoblastoma binding protein 6 (RBBP6) is overexpressed in esophageal, lung, hepatocellular and colon cancers [1,2,3,4]. Germline mutations, mostly in the p53 binding domain of RBBP6, are reported to cause predisposition to familial myeloproliferative neoplasms [5], thus implicating a p53-dependent pathway in pathogenesis. Furthermore, RBBP6 was identified as an independent prognostic marker for overall and for disease-free survival in colon cancer patients [3]. Taken together, these findings indicate that RBBP6 may be an important target for anti-cancer drugs, as well as a candidate diagnostic marker.

The RBBP6 family is found in eukaryotes, but not in prokaryotes, and orthologues are known by different names. The human protein is known as RBBP6, the mouse as PACT or P2P-R [6,7], the fruit fly as SNAMA [8] and the worm as RBPL-1 [3]. Generally, in vertebrates, *RBBP6* produces multiple isoforms by alternative splicing or from two promoters, as predicted in the case of the human gene [9]. These isoforms consist of the common domain with no name (DWNN), whose tertiary structure resembles that of ubiquitin together with various combinations of other domains and motifs [10,11]. Interestingly, the shortest isoform, which comprises the DWNN only is downregulated in human cancers, while the larger isoforms tend to be upregulated [1]. Essentially, this isoform comprises the DWNN, whose tertiary structure resembles that of ubiquitin [8,12]. A possible explanation for this may be found in new evidence showing that DWNN, as an independent module, antagonizes the larger isoforms by competition. This was evident when overexpression of this isoform resulted in inhibition of the 3' end pre-mRNA cleavage in a manner similar to siRNA-mediated knockdown of the full-length RBBP6 [13].

SNAMA is essential for embryonic development and appears to suppress cell death, as its deletion results in the abnormal occurrence of apoptosis during embryogenesis. Under normal physiological conditions, maternally contributed *Snama* transcripts decline in the embryo six hours into development, and adult expression is reduced in males when compared to females [8]. In the developing eye, *Snama* is required for cell proliferation and for cell survival anteriorly to the morphogenetic furrow and is regulated by hedgehog signaling. Furthermore, SNAMA controls nucleic acid metabolism profoundly [14]. Although the molecular interactions of the invertebrate orthologues are not known, some insight may be gained by observing the vertebrate genes. The PACT protein was shown to act by enhancing Mdm2 activity and to exhibit a similar phenotype to Mdm2. The *Pact^−/−^* phenotype is lethal and can be partially rescued by simultaneous deletion of p53. However, the *Pact^−/−^ p53^−/−^* phenotype is more severe than that of *mdm2^−/−^ p53^−/−^*. Notably, these mutant mice develop a distinct anterior-posterior pattern [15], indicating that *PACT* may be involved in embryonic patterning.

SNAMA and RBPL-1 are similar to the vertebrate counterparts in sequence features, but the absence of an *Mdm2* homologue is profoundly enigmatic and has attracted scrutiny. This led to a conclusion that a *Mdm2* homologue eluded the fruit fly and the worm [16]. Nevertheless, this status makes *Drosophila* and *C. elegans* attractive models for studies of Mdm2-independent functions of the RBBP6 family members.

The invariable DWNN occurs independently or in combination with other domains and motifs, such as the p53-binding and pRB-binding domains, the RING-finger and zinc finger motifs. In addition there may be combinations of features, like proline-rich, lysine-rich, glutamic acid-rich or SR-containing regions or a nuclear localization signal [12]. It has been postulated, based on sequence comparisons, that SNAMA has a p53-binding domain [10], but this has not been demonstrated experimentally. Furthermore, the existence of a single *Snama* transcript was assumed until a second and shorter isoform was predicted by bioinformatics analyses. This is confirmed experimentally in the present study.

The *C. elegans* RBPL-1 is essential for embryonic and germline development and for nutrient synthesis in the intestines. Interestingly, silencing RBPL-1 causes dramatic changes in the expression of more than 700 genes [17]. These data suggest multiple roles of the RBBP6 family in biological systems.

Overall, the abovementioned facts indicate that RBBP6 proteins are nuclear proteins, which possess E3 ligase activity through the RING finger, are probably involved in pre-mRNA processing, including splicing and 3' polyadenylation cleavage, and act as part of macromolecular complexes. Moreover, RBBP6 proteins are localized in the nucleoli of interphase cells and at the periphery of chromosomes in mitotic cells, which lack nucleoli [18]. This is supported by their detection in nuclear speckles and their tendency to co-immunoprecipitate with nuclear matrix-associated proteins. Human RBBP6 also regulates DNA replication together with the *Drosophila Krüppel* homolog, ZBTB38 and MCM10. Its absence slows down replication fork progression, causing complete disruption of common fragile sites, which are the sites most susceptible to replication stress [19]. Thus, the accumulated data so far indicate that RBBP6 plays crucial roles in animal physiology, making it a druggable candidate.

The present study confirms the existence of two *Snama* transcripts that are differentially expressed during embryogenesis. The putative promoter region is found to have potential binding sites for transcription factors that are required for anteroposterior axis formation, segment polarity during embryogenesis and for cell proliferation and differentiation. Moreover, putative binding sites for the key transcription factor for cell proliferation proteins, known as the DNA replication-related element factor (DREF), and for p53 are predicted.

## 2. Results

### 2.1. Transcriptional Start Site Mapping by 5' Rapid Amplification of cDNA Ends and Functional Analysis of the Promoter Region

**Figure 1:**
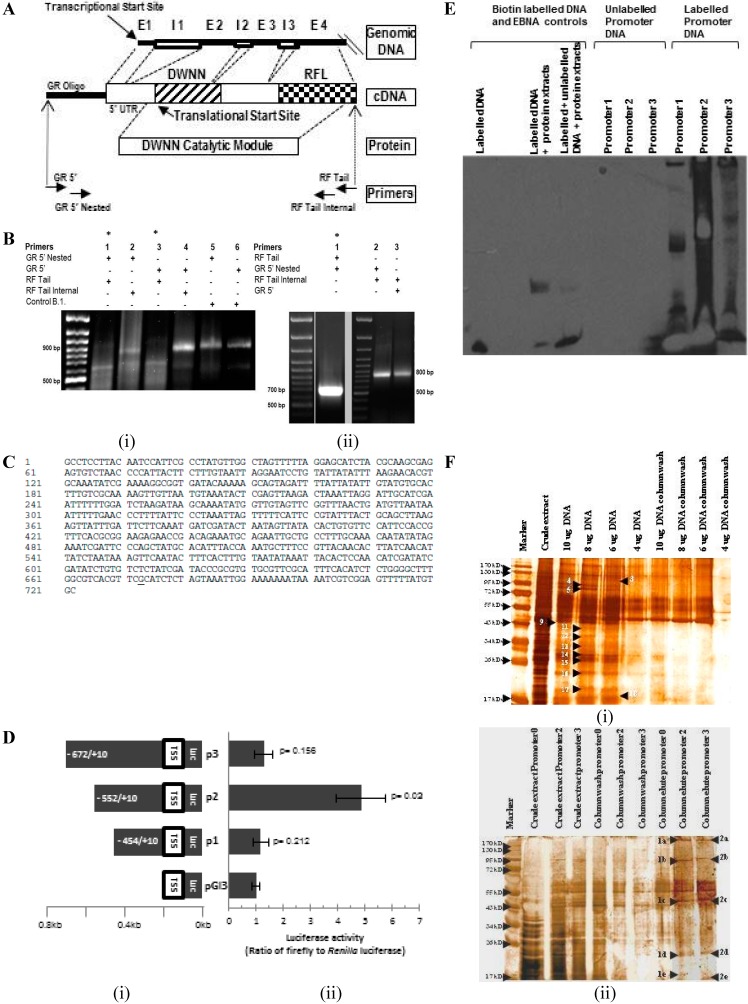
Analysis of the putative *Snama* promoter. (**A**) Schematic representations of the *Snama* genomic DNA starting at the putative transcriptional start site (TSS), the cDNA expected from rapid amplification of cDNA ends (RACE) and the domain with no name (DWNN) catalytic module. Arrows indicate primers used as specified in the Materials and Methods; E = exon, I = intron (**B**) 5' RACE products using *Drosophila* mRNA: (i) Amplification using combinations of GeneRacer 5' nested and GeneRacer 5' forward primers together with the RING finger (RF) tail and RF tail internal as reverse primers gives rise to a ~800-bp product (Lanes 2 and 4), and a smaller product of ~650 bp (indicated by an asterisk) was amplified when the GeneRacer 5' nested and GeneRacer 5' forward primers are combined with RF tail reverse primer (Lanes 1 and 3). A positive control showing the amplification of the RACE-ready β-actin cDNA, created from HeLa RNA, results in a ~900-bp fragment, as expected (Lanes 5 and 6). The difference in size between the β-actin cDNA amplification product with GR 5' nested primer (858 bp) and GR 5' primer (872 bp) is only 14 bp; (ii) Cloning and PCR amplification of the 5' RACE products: Lane 1 shows the smaller ~650-bp cloned fragment (asterisk), whilst Lanes 2 and 3 show the larger ~800-bp cloned fragment; (**C**) Sequence of the putative promoter region. The underlined nucleotide is the putative transcriptional start site; (**D**) (i) Schematic representation of fragments (P1–P3) of the promoter region showing base positions relative to the transcription start site (+1); (ii) Dual luciferase assay to determine the maximal promoter sequence. Measurement of luminescence activity of firefly and *Renilla* luciferase activity indicated that the maximal promoter sequence required to drive *Snama* transcription was Promoter 2; (**E**) Mobility shift assay of biotin-labelled and unlabeled DNA Snama promoter DNA. Lanes 1–3 indicate the Epstein–Barr nuclear antigen (EBNA) control system, biotin-EBNA control DNA, biotin-EBNA control DNA and EBNA extract and biotin-EBNA control DNA and EBNA extracted with excess unlabeled EBNA DNA, respectively. The biotin-EBNA control DNA shows no shift; the biotin-EBNA control DNA and EBNA extract shows a shift; and the biotin-EBNA control DNA and EBNA extract and excess unlabeled EBNA DNA show minimal shift due to competition. Lanes 4–6 show the unlabeled DNA fragments P1, P2 and P3. Lanes 7–9 represent labelled promoters P1, P2 and P3 and a mobility shift due to the binding of nuclear proteins to the promoters; (**F**) Electromobility shift assay (EMSA) of biotin-labelled protein-DNA complexes separated from crude extracts by streptavidin affinity chromatography. Promoter 0 represents the −231 region. (i) Increasing concentrations of labelled Promoter 2 DNA were used to elute binding proteins from embryonic nuclear extracts; (ii) Four micrograms of promoter DNA as indicated were used in order to select the best promoter to use in (i). Arrows indicate the bound nuclear proteins that were selected for identification by mass spectrometry; (**G**) Schematic representation of the (−673; +49) sequence shows the predicted transcription factor binding sites.

To identify the transcriptional start site (TSS) of *Snama*, 5' rapid amplification of cDNA ends (5' RACE) was conducted employing primers as indicated by the arrows in Figure 1A. The forward primers, GR 5' and GR 5' nested, are based on the GeneRacer™ RNA oligonucleotide that is ligated to the 5' end of the full-length and decapped mRNA. The primer combinations produced two DNA fragments of about 650 and 850 base pairs long (Figure 1B). The fragments were cloned into pGEM^®^ T Easy (Figure 1C) and sequenced. Both sequences were aligned with themselves and with the *Snama* genomic sequence containing the putative regulatory region to identify the transcriptional start site (TSS) (underlined in Figure 1C). The smaller band was ignored, as it is derived from an unrelated gene 4.6 Mbp upstream of *Snama*, in the right arm of chromosome 2 of *Drosophila*, due to similarity with the RING finger (RF) tail primer. The RF tail primer used during 5' RACE was shown to be partly homologous with a gene upstream of *SNAMA*. Thus, the primer bound to both the *SNAMA* gene and the gene upstream, which were then both reverse transcribed and amplified. This also accounts for the virtual absence of this fragment when 5' RACE was performed using the RF tail internal primer (Lanes 2 and 4 in Figure 1B(i)).

The *Snama* gene is flanked by CG16786 located approximately 500 bases upstream of the TSS and by *genghis khan* (*gek*) downstream and constitutes the putative regulatory region. We conducted the luciferase reporter assays using a series of recombinant plasmid constructs containing fragments of this region (Figure 1D) to show that this region can drive transcription. Promoter region 2 (−552) elicits the strongest activation of transcription. We therefore selected P2 and P3 to perform the electromobility shift assay (EMSA) to show that this region can bind cellular proteins. The observed stronger intensity of the signal in P2 suggests that more proteins bind to this fragment compared to P3. The biotin-labelled P2 and P3 fragments were also used to capture nuclear proteins on streptavidin agarose beads for identification by mass spectrometry. The eluted proteins were selected for mass spectrometry by comparison with proteins that were released during column washing (Figure 1F, Table 1). A number of proteins, including several nuclear proteins, including the DNA replication-related element factor (DREF), PHAX, heat shock protein 27 and hsp83 (hsp90) and the transcriptional co-activator pyruvate kinase, was identified by MS.

The sequence of the putative promoter region was used to search the Transfac database for insect transcription factors (TFs) using the Patch program. This revealed the set of potential TF binding sites shown in Figure 1G and summarized in Table 2. The predicted transcription factors include proteins involved in embryonic patterning, cell proliferation, cell differentiation, germline development and in sex determination. Furthermore, the predicted factors associated with embryonic patterning appear to be proteins that mediate the anteroposterior axis formation. The potential binding sites for hunchback, two for caudal and one for bicoid were detected.

Many proteins that are regulated by p53 often contain p53 DNA-response elements (p53REs), and mammalian RBBP6 proteins are known to interact with p53. We therefore investigated the putative promoter region for p53REs. The consensus sequence for the p53 response elements is degenerate and consists of two repeats of sequence 5'-PuPuPuC(A_T)-3', where (T_A)GPyPyPy is its inverted version. These 10-base palindromic sequences often occur in pairs separated by a spacer sequence. The p53 protein binds as a tetramer to this sequence. While the degeneracy of the p53 binding sequence might allow efficiency when responding to a diversity of cellular signals and is therefore desirable, it is complex and is not predicted by many bioinformatics programs. Therefore, we used the p53MH program [20] to search potential p53REs. We found a potential site at position-654 (5'-TGGCTAGTTT... TTAGGAGCATCT ...ACGCAAGCGA-3') with a high score (79).

**Table 1:** Putative promoter-binding proteins (EMSA). Proteins were analyzed by MS/MS and by the ProteinPilot software, as described in the Materials and Methods. ProtScore is a relative score for determining and comparing the overall coverage of a protein. The score is based on the cumulative confidence scores of all peptide fragments detected for an individual protein. A score of ≥2 represents ≥99% confidence, while a score of ≥1 is representative of ≥90% confidence.

Band	MS Prediction	Accession Number	Size (AA)	ProtScore
**TRANSCRIPTION FACTORS**				
4	DREF transcription factor	O96083	709	2.03
**TRANSCRIPTION CO-ACTIVATOR**				
2c	Pyruvate Kinase	AAO24935		2
**OTHER NUCLEAR PROTEINS**				
5	Heat shock protein 83	P02828	717	1.66
14	Heat shock protein 27	HHFF27	213	2
17	Ribosome biogenesis protein WDR12 homolog	Q9VKQ3	420	1.11
	Similar to PHAX	Q8SYG4	479	1.17
**PROTEINS ASSOCIATED WITH PRE-MRNA PROCESSING**				
3	CG4266	Q9W2K4	1215	2
**OTHER PROTEINS**				
1a	Mind-meld	CG9163-PB Q7KUY7	783/840	0.93
1c	With coiled coil and lipase domain	Q29QE3, GH19966p	482	2
1d	elongation factor 1alpha48D	AAF58608		15.56
	Actin 87E-fruit fly	S04538		1.92
		Q9VEV0, CG10405-PA	252	0.8
1e	Unknown (CG17374)	Q7PLB8	2284	2
	Tubulin	Q9V7Y7,CG15611	508	1.6
2a	Unknown	Q9VGU9	332	2
2c	CG9277-PA (Tubulin family)	Q9V8V3		10
	ATP synthase subunit β	Q05825.3	505	3.2
2d	Unknown	Q9VLX3	281	1.26
	Hypothetical protein	Q9NG77	81	1.06
		Q7KQM6		0.74
2e	Unknown	Q9VU21	308	2.04
	Unknown	Q4ABH1	11,707	0.77
	Unknown	Q9VQ84	124	2
9	CG18063-PA, isoform A	Q9VJM6	337	0.9
11	Unknown	Q1RKR2	270	2
		Q9VEJ9	753	0.84
13		Q9VP46		1.05
16		Q9VJM6/ CG18063-PA	337	0.75
17	Unknown	Q9VQ84	124	1.64
18	With the Domain of Unknown Function and coiled coil	Q9VQ83	559	1.52

**Table 2:** Putative transcription factor binding sites on the *Snama* promoter region.

Group	Genes	References
Embryonic patterning	Caudal, bicoid, tailless, hunchback	[21,22]
Segment polarity	Engrailed, LEF-1 (pangolin)	[23,24]
Regulation of development	Adf-1 (Myb)	[25]
Germ-line development and sex determination	dsxf/dsxm, sry- = β, CF-2	[26,27,28]
Replication	DREF	[29]
Myogenesis	MEF2, twist, CF2	[27,30,31,32,33]
Chromatin structure	BEAF, GAGA	[34,35,36]

### 2.2. Differential Expression of Snama during Drosophila Development

**Figure 2:**
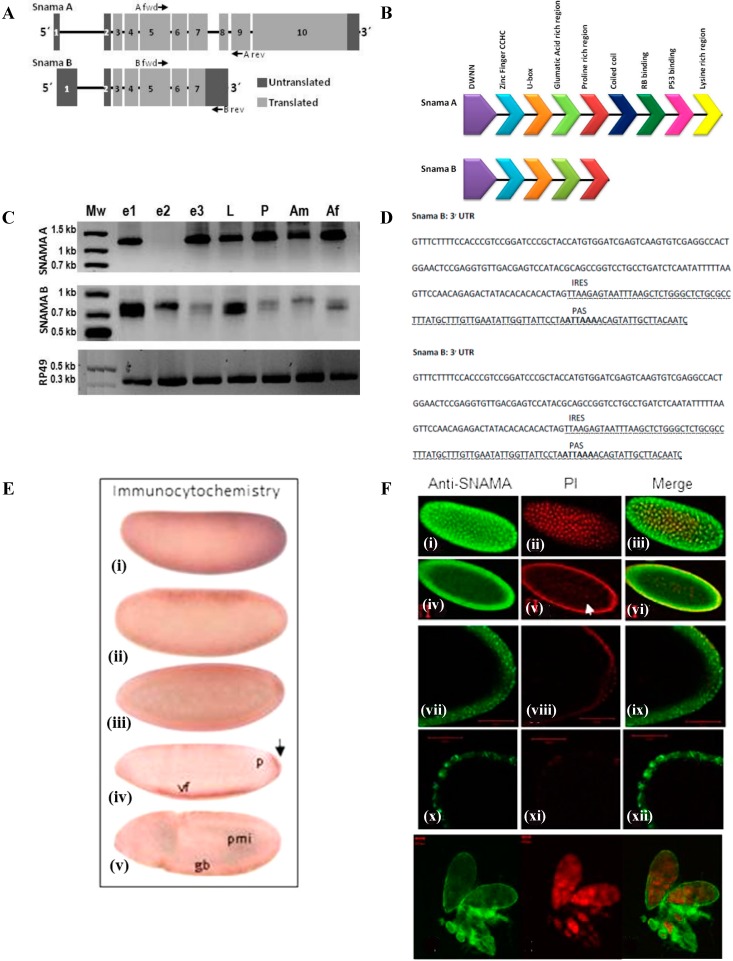
Differential expression of SNAMA during oogenesis and embryogenesis. (**A**) Schematic representation of the Snama genomic organization showing the exon structure of *Snama* A and B transcripts; (**B**) domain structure of SNAMA A and B proteins; (**C**) differential expression of the SNAMA isoforms determined by RTPCR on total RNA obtained from embryos e1 (0–3 h), e2 (3–6 h) and e3 (6–9 h), larvae (L) pupae (P), adult males (Am) and adult females (Af); (**D**) an analysis of the untranslated regions of *Snama* A and B using the UTRScan program (Available online: http://itbtools.ba.itb.cnr.it/); (**E**) (i–v) spatial expression of SNAMA determined by immunocytochemical staining of embryos with anti-DWNN antibody. Pole cells are indicated by an arrow. vf indicates the ventral furrow and gb the germ band; (**F**) Subcellular localization of SNAMA protein during the blastoderm stage by immunocytochemical staining with anti-DWNN antibodies and viewed by confocal microscopy. (i–vi) Expression of SNAMA (green) and nuclei (red) during blastoderm embryo and a merged imaged of the two; (iv–vi) expression of SNAMA and nuclei and a merged image during the cellular blastoderm stage; (vii–xii) a magnified view of the posterior end of an embryo showing punctate staining; (xiii–xv) localization of SNAMA in an ovariole.

A recent update of the Flybase genome database predicted a second *Snama* transcript, referred to here as *Snama B*. These transcripts have distinct 5' UTRs (untranslated regions) and share exons 1 through 7. The shorter isoform, called *Snama B*, is generated by alternative splicing. Consequently, *Snama A* consists of three additional exons, as shown in Figure 2A, and encodes a protein with multiple domains, including the p53 and Rb-binding domains in Figure 2B. Based on the exon structure of the two transcripts, primers that can distinguish between them were designed as indicated in Figure 2A. Reverse transcriptase polymerase chain reaction (RT-PCR) performed on mRNA obtained from various stages in the life cycle of the fly and performed on three independent experiments indicates that the two transcripts are maternally provided and are regulated differentially during development (Figure 2C). RT-PCR was performed on equal amounts of cDNA for each category of sample, so that differences in abundance of transcripts may be discerned semi-quantitatively. Although both transcripts are present in the early embryo, it is notable that *Snama A* is not detectable in the window representing 3–6 h embryos (e2) in several independent experiments. This probably reflects that the maternally-derived *Snama B* transcripts persist longer. On the other hand, the maternally-contributed *Snama A* transcripts are depleted within the first three hours of development. They are then replenished by zygotic transcription and persist abundantly throughout the life cycle of the fly.

Since the two transcripts differ appreciably in their untranslated regions (UTRs), we used the UTRScan program (Available online: http://itbtools.ba.itb.cnr.it/) to evaluate them for conserved regulatory patterns (Figure 2D). *Snama B* has a larger 5' prime UTR containing three upstream open reading frames (uORFs). There are no plausible patterns detectable in the *Snama A* 5' prime UTR. Upstream open reading frames provide another level of posttranscriptional control of gene expression and are found to be particularly common in oncogenes and in genes involved in cellular growth and differentiation [37]. Unlike *Snama B*, *Snama A* has a cytoplasmic polyadenylation element (CPE) UUUUUAAU in the 3' prime UTR alongside the ubiquitous hexanucleotide polyadenylation signal (PAS) AAAUAA that is crucial for nuclear pre-mRNA cleavage and polyadenylation. The two *cis* elements are 31 nucleotides apart. This suggests that *Snama A* has a mechanism for translational control, as CPEs are found in dormant cytoplasmic mRNA that can be activated by specific signals [38]. CPE has recently been demonstrated in *Drosophila* [39]. However, *trans* factors are poorly known.

We used an anti-DWNN antibody that does not cross react with SR-protein to determine spatial expression during embryogenesis. Although this antibody cannot distinguish between the two SNAMA isoforms, the staining pattern shows that SNAMA is expressed abundantly and ubiquitously in the blastoderm and reaches lower levels by the cellular blastoderm. However, by this stage, higher concentrations are localized in the ventral furrow and in germ cells (Figure 2E).

We also used immunofluorescent staining and confocal microscopy to visualize the localization of the SNAMA protein in the blastoderm. SNAMA is present in all of the synchronous pre-blastoderm stage nuclei, but later, it is found in the cortical nuclei and absent in the mitotically inactive “yolk” nuclei (Figure 2F). Similarly, SNAMA is not expressed in nurse cell nuclei where endoreplication is known to occur during oogenesis (Figure 2F). It is notable also that nuclear staining is evident in mitotically dividing cells and not in endoreplicating syncytial blastoderm nuclei. Overall, the staining patterns observed by immunocytochemistry and confocal microscopy are both punctate and diffuse, suggesting cytoplasmic, as well as nuclear localization.

### 2.3. Subcellular Localization of SNAMA and Potential Interacting Partners

**Figure 3:**
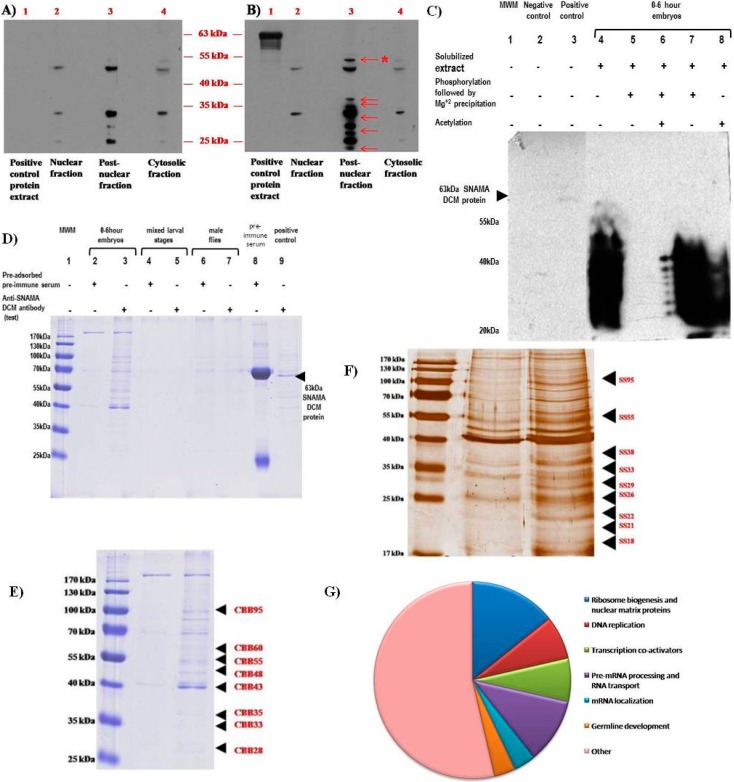
Subcellular localization of SNAMA and detection of interacting proteins. (**A**–**C**) Immunoblot analysis of fractionated sub-cellular extracts obtained from 0–6-h-old *D. melanogaster* embryos; two identical blots were probed with either (**A**) pre-adsorbed pre-immune serum or (**B**) anti-SNAMA DCM antibodies. Six unique bands are detected in Blot B (red arrows) and are all present in the post-nuclear fraction. The band with an asterisk matches the size of SNAMA B; (**C**) Immunoblot of acetylated protein extracts from 0–6-h *D. melanogaster* embryos. Samples were subjected to acetylation, phosphorylation and/or a selective precipitation procedure, as shown, and then detected with anti-SNAMA DCM antibodies. The SR-precipitated pellet fraction was re-suspended in HEPES buffer, prior to acetylation. The blot shows detection of a “ladder” of low molecular weight proteins. These are likely serine/arginine SR-proteins detected by the characteristic precipitation reaction for SR-proteins. SNAMA A is not detected, as expected. SNAMA B would not be detected in this procedure, since it is not an RS-domain containing protein. The negative control contains a bacteria containing the recombinant GST tag, and the positive control consist of the purified GST-SNAMA DCM fusion protein; (**D**–**F**) SDS-PAGE analysis of immune-complex proteins isolated from co-immunoprecipitation assays; (**D**) samples obtained from 0–6-h embryos (Lanes 2 and 3), combined larval stages (Lanes 4 and 5) and male-only flies (Lanes 6 and 7). For each stage of development, a negative control assay was performed (Lanes 2, 4, 6). The pre-immune serum was also included to exclude residual serum proteins when selecting bands for further identification (Lane 8). Equivalent bands between both test and control lanes, for a given stage of development, were negated. Unique bands were detected exclusively in the embryo sample (Lane 3); Lanes 2 and 3 in (**D**) were stained with Coomassie blue (**E**) and with silver stain (**F**), and bands indicated by arrows were identified by mass spectrometry; (**G**) Representation of the relative percentages of transcription factors identified by tandem mass spectrometry as grouped by their biological function.

We analyzed 0–6-h embryonic extracts by Western blot using the chicken anti-SNAMA DCM polyclonal antibody. Aware that a small set of proteins are recognized by the pre-adsorbed pre-immune serum, indicating non-specific recognition (Figure 3A), we focus on the proteins that are detected exclusively by the anti-SNAMA DCM antibody (Figure 3B arrows), which all localize to the post-nuclear fraction. With the exception of the 55-kDa band, the remaining bands are likely to represent arginine-serine(RS)-domain-containing proteins (Figure 3C, Lane 6), because they can be precipitated by MgCl^2+^ and then re-dissolved in high salt with EDTA [40], a characteristic reaction for RS-domain-containing proteins. Antibodies to the orthologous mouse PACT are known to also cross-react with RS-domain-containing proteins [6], probably due to sharing epitopes with similar regions downstream of the DWNN. However, the approximately 55-kDa band indicated by an asterisk in (Figure 3B) is likely to be SNAMA B, because it is not detected by the pre-immune serum (negative control) (Figure 3A) and matches the expected size. Furthermore, this band is also present in the untreated extract in (Figure 3C, Lane 4), but is absent from the fractions of the treated sample (Lanes 6 and 7), which select for RS-domain-containing proteins, a domain that the shorter SNAMA B lacks. SNAMA A was not detected in all fractions, probably due to low expression levels. It was expected that SNAMA A would be present in the nuclear fraction, since it has a nuclear localization signal and is shown by immunocytochemistry to be associated with chromatin elements. However, it is likely that it occurs at levels that are undetectable by Western blot analysis.

To identify proteins that interact directly or that associate in multi-molecular complexes with SNAMA, we used crude extracts and anti-SNAMA DCM antibodies to create immune complexes and captured them on affinity columns, as indicated in Figure 3D. A pre-adsorbed pre-immune serum was used as a control to exclude non-specifically bound proteins. Because a distinct set of proteins was conspicuous in the 0–6-h embryo extract, this sample was chosen for identification of the proteins by mass spectrometry. The gels were stained by Coomassie Blue and by silver staining, and bands were selected for sequencing by tandem mass spectrometry, LC-MS/MS (Figure 3D–F). Mass spectrometry revealed proteins that are implicated in ribosome biogenesis, DNA replication, RNA transport, pre-mRNA splicing and transcription co-activators (Figure 3G; Table 3). Interestingly, some proteins that were detected in the EMSA were also identified in these co-immunoprecipitation experiments, indicating nucleic acid-dependent and -independent associations. These proteins include the DNA replication-related element-binding factor (DREF), heat shock protein 27, the phosphorylated adaptor for RNA export (PHAX) protein that transports U3 snoRNA from the nucleus after transcription, CG4266 and the ribosome biogenesis protein WD12 homolog. In addition to their involvement in apoptotic pathways (nucleic acid-independent process), RBBP6 family members have been shown to be part of large multi-protein complexes involved with pre-mRNA 3' end cleavage and polyadenylation, as well as in pre-MRNA splicing [13]. This implies that they form nucleic acid-associated protein complexes at the carboxy-terminal domain of RNA polymerase II and at spliceosomes.

**Table 3:** Proteins that are co-immunoprecipitated with anti-SNAMA antibodies.

Sample ^1^	UniProt Accession Code	Name	Molecular Weight (kDa)	ProtScore
CBB55	Q9TWZ1_DROME	D-ERp60 (PDI)	55.373	2.26
CBB35	Q9UAN1_DROME	60S RIBOSOMAL PROTEIN L22	30.611	2
CBB43	RL4_DROME	60S RIBOSOMAL PROTEIN L4	45.026	2.37
SS95	Q9VKQ3_DROME	RIBOSOME BIOGENESIS PROTEIN WDR12 HOMOLOG	47.222	1.47
SS22	Q94883_DROME	DNA REPLICATION-RELATED ELEMENT FACTOR (DREF)	80.727	1.24
SS95	HP1_DROME	HETEROCHROMATIN PROTEIN 1	23.185	1.31
TRANSCRIPTION CO-ACTIVATORS				
CBB90	PYG_DROME	GLYCOGEN PHOSPHORYLASE	96.997	2
CBB55	KPYK_DROME	PYRUVATE KINASE	57.44	2.75
SS18 and CBB33	Q9W2K4_DROME	CG4266-PA	130.427	1.74 and 0.89
CBB28	HSP27_DROME	HEAT SHOCK PROTEIN 27	23.617	4
SS95	Q8SYG4_DROME	PHOSPHORYLATED ADAPTER FOR RNA EXPORT (PHAX)	53.965	1.17
SS18 and SS26b	Q9W1K4_DROME	EGALITARIAN	112.129	1.43 and 1.15
SS18, SS21 and SS22	Q24156_DROME	STONEWALL	112.913	0.87, 0.94 and 1.08
CBB28	VDAC_DROME	VOLTAGE-DEPENDENT ANION-SELECTIVE CHANNEL	30.55	8.13
CBB43	VIT2_DROME	VITELLOGENIN-2	49.66	14.35
CBB55	TBB1_DROME	TUBULIN Β-1 CHAIN	50.147	16.55
CBB90	TERA_DROME	TRANSITIONAL ENDOPLASMIC RETICULUM ATPASE TER94	88.859	9.72
SS95	Q9W003_DROME	SPINOPHILIN	233.209	1.86
SS22d	Q4ABH1_DROME	MUSCLE-SPECIFIC PROTEIN 300, isoform D	1405.148	0.97
SS33	Q9VXL1_DROME	MIND-MELD, isoform C	150.547	0.87
CBB28	ADT_DROME	ADP, ATP CARRIER PROTEIN	34.215	5.64
SS95b	Q6IGH5_DROME	HDC06258	17.442	0.78
SS22	Q9VQ83_DROME	CG10874-PA	35.041	1.57
SS38	Q9VSY1_DROME	CG4022-PA	57.294	2
SS26	Q4V5H1_DROME	PEPTIDYL-PROLYL CIS-TRANS ISOMERASE	20.182	1.62
CBB90	EF2_DROME	ELONGATION FACTOR 2	94.459	27.74
CBB43	EF1G_DROME	ELONGATION FACTOR 1-GAMMA	48.968	6
SS29	EF1A2_DROME	ELONGATION FACTOR 1-ALPHA 2	50.663	2.5

## 3. Discussion

RBBP6 is emerging as an attractive candidate for the development of anti-cancer drugs due to its role in cell proliferation and altered expression patterns associated with certain cancers. We have confirmed the existence of a shorter isoform of SNAMA and provide evidence that both isoforms are developmentally regulated. Promoter analysis suggests that RBBP6 may be involved in embryonic anteroposterior patterning and that it may be under the control of the DNA replication element, which is known as the “master key” for cell proliferation [29]. Interesting phenotypes observed in mammalian models indicate crucial roles played by this gene in patterning of the developing organism and in malignant pathologies. These data suggest that RBBP6 proteins are important players in carcinogenesis and in developmental defects.

The regulatory region of *Snama* extends over approximately 400 bases upstream of the transcriptional start site. The theoretically-determined TSS in Flybase is located 37 nucleotides upstream of the position experimentally-determined in the current study. We used the GeneRacer™ method, because it promises full-length mRNA and elimination of truncated transcripts.

Bioinformatics search reveals potential sites for binding of transcriptional factors that regulate cell proliferations, patterning along the anteroposterior axis, mesoderm development and sex determination. Notable amongst the proteins identified by EMSA, co-immunoprecipitation and MS were transcription-related proteins, such as the DNA replication element factor (DREF), CG4266 and heat shock protein 27 (hsp27). DREF is recognized as the “master key” for the coordinate expression of cell proliferation-related genes. It binds to the transcription regulatory DRE (5'-TATCGATA) on promoters of proliferation-related genes. Furthermore, DREF is downregulated by distinct mechanisms during differentiation, suggesting concomitant repression of cell proliferation via the DREF pathway [29]. DREF can also bind competitively to an overlapping sequence recognized by boundary element-associated factor (BEAF) [41]. It plays an important role in DNA replication, probably by acting on key molecules at the replication fork and on key elements of the chromatin. Ectopic expression of DREF targeted at the post-mitotic cells behind the eye morphogenetic furrow results in increased DNA content, apoptosis and a rough eye phenotype [42]. This is reminiscent of the role played by SNAMA at the eye morphogenetic furrow [14]. The detection of DREF in the band-shift assay, as well as in co-immunoprecipitation hints at the possibility that SNAMA is transcriptionally regulated by DREF and also interacts with it directly or indirectly in protein complexes. It is not unusual for transcription factors to be regulated by their targets in feedback loops. CG4266 contains the arginine- and proline-rich (RPR) and the RNA recognition (RRM) motifs typical of proteins that link gene transcription to pre-mRNA processing (capping, splicing and polyadenylation). Generally, macromolecular complexes, of which SNAMA could be part, recognize the carboxy-terminal domain of RNA polymerase II to enable pre-mRNA processing at the 5' end, as well as at the 3' end [43]. CG4266 may be a component of such complexes. The hsp27 protein is expressed in the absence of heat shock during *Drosophila* development and exhibits complex expression patterns during development, indicating multiple roles [44]. Hsp27 also associates with nuclear speckles independently of the nuclear localization signal, suggesting nucleo-cytoplasmic shuttling that is mediated by active transport [45]. The association of *Snama* with various aspects of nucleic acid metabolism and cell cycle control matches documented roles played by RBBP6 proteins. Although SNAMA possesses putative E3 ligase activity, other enzymes involved in the ubiquitination system, such as the E1 ubiquitin-activating enzyme and the E2 ubiquitin-conjugating enzyme, were not detected by co-immunoprecipitation. This is probably because E3 ligases bind to their substrates and only make transient catalytic associations with the components of the enzymatic pathway, particularly the E2-ubiquitin complex.

The biological roles of some of the multiple isoforms of mammalian RBBP6 have been characterized to some extent. For example, isoform 3 of human RBBP6, which is essentially DWNN, is reported to control the cell cycle at G2/M transition, since reduced expression leads to fewer cells at G2/M. Alternatively, RBBP6 is induced exclusively during the G2/M transition [1]. SNAMA B, which is structurally closest to RBBP6 isoform 3, is expressed at higher levels during the blastoderm when the syncytial embryo undergoes the S/M cell cycle and not the full cell cycle. Unlike the RBBP6 isoform 3, SNAMA B has a C-terminal extension with zinc finger and U-box motifs, but lacks the p53 and Rb binding domains. *Drosophila* embryonic development is characterized by discrete spatiotemporal waves of mitosis. In the pre-blastoderm, there are rapid and synchronous divisions of the nuclei comprising cycles of DNA synthesis (*S*-phase) and mitosis (M) without gap phases. Later, during the cellular blastoderm stage, there are mitotic domains that are coordinated in elaborate spatiotemporal patterns [46]. In the interior of the cellular blastoderm embryo, endoreplicating cells are found. These cells undergo repeated S-phases without mitosis. The present study shows that SNAMA is expressed in the periphery of the cellular blastoderm, but not in the endoreplicating cells at the interior of the embryo. SNAMA is also absent in the endoreplicating nurse cells. Taken together, these results suggest that SNAMA proteins are required for mitosis, but not for replication of DNA.

The prediction of binding sites for caudal, bicoid, hunchback and others suggests that *Snama* may be involved in anteroposterior (A-P) embryonic patterning, since these proteins are known to regulate the establishment of the A-P axis. These findings are reminiscent of the phenotype observed when the mouse PACT/P2P-R is perturbed. *pact^−/−^* mice die during embryogenesis with widespread apoptosis. This phenotype can be partially rescued by the concomitant deletion of p53 [15]. Similarly, *mdm2* null mutants die at the implantation stage (around 5.5 days of development). This phenotype can be rescued by concomitant deletion of p53, resulting in viable offspring, albeit with subtle defects [47]. While these phenotypes are similar, the *pact^−/−^* mice appear to have more severe defects, indicating that PACT may have additional functions to promoting the activity of Mdm2, as suggested by Li *et al.* [15]. These partially rescued *pact^−/−^* embryos are characterized by a distinct anterior-posterior pattern, suggesting an important role for PACT in mouse embryonic patterning. The involvement of RBBP6 in embryonic development therefore requires careful attention, because lesions that affect developmental patterns are of clinical significance, as they may be associated with birth defects.

The expression pattern produced by immunocytochemical staining suggests that SNAMA is upregulated in germ cells (pole cells). We also predicted potential binding sites for germline and sex determination transcription factors by bioinformatics analysis. Furthermore, some of the transcription factors detected by co-immunoprecipitation and EMSA provide further support for the involvement of SNAMA in germ-line development and in sex determination. Firstly, the RNA-binding maternal protein Egalitarian (Egl) (Table 3) is required for oocyte determination in complex with bicaudal (BicD) [48,49]. Secondly, co-IP experiments identified Stonewall, a transcription factor that is structurally similar to Adf-1 (Table 2) and known to be involved in germ cell development [50,51].

In *Drosophila*, sex determination is controlled by a hierarchy of sex-specifically spliced genes culminating in the production of the terminal genes *doublesex* (*dsx*) and *fruitles*s. The pre-mRNAs transcribed from these genes are sex-specifically spliced to produce *DSX^F^* for females and *DSX^M^*, whose binding sites on the *Snama* promoter are predicted here. In females, the hierarchy is initiated by sex lethal (Sxl), which regulates the alternative splicing of *transformer* (*tra*). Tra then regulates the alternative splicing of *dsx* to *Dsx^F^*. In males, where S*xl* is absent, *dsx* is alternatively spliced by default to *Dsx^M^* and *Fru^M^* for males. Thus, the *dsx* pre-mRNAs are expressed differentially between the two sexes and determine somatic differentiation, whereas FRU controls behavior associated with male courtship [52]. Amongst *Dsx^F^* binding sites identified in 650 regions of the *Drosophila* genome, one is near CG16786. [26]. This gene, whose function is unknown, is located immediately upstream of *Snama*. The prediction of Serendipity (sry-β) binding sites provides evidence for potential involvement of SNAMA in germ-line development [53]. The presence of CF2 binding sites suggest that SNAMA is involved in follicle cell signaling during oogenesis [28].

## 4. Experimental Section

### 4.1. Maintenance of Fly Stocks

Wild-type *Drosophila melanogaster* (Canton S), were reared at 25 °C on apple juice agar plates with yeast.

### 4.2. RNA Extraction and RT-PCR

Total RNA was collected from all samples by using TRI Reagent^®^ (Sigma-Aldrich, St Louis, MO, USA) following the manufacturer’s protocols. As the first step to PCR, complementary DNA (cDNA) was synthesized according to the protocol described in the Thermo Scientific RevertAid First Strand cDNA synthesis kit using specific (reverse) primers, as described below. Further, to distinguish between the two Snama transcripts, the cDNA was amplified by PCR using specific primers as follows: SNAMA A Fwd: 5'-TGAGGAATTCGGTGAACG-3', Rev 5'-CAACGGATCGTCAATGG-3'; and SNAMA B (Fwd): 5'-TGAGGAATTCGGTGAACG-3', Rev 5'-ATCCACATGGTAGCGGAT-3'. RT-PCR was performed using the DreamTaq PCR master mix according to the manufacturer’s protocol (Thermo scientific, Waltham, MA, USA) with appropriate adjustments for the primers. As a loading control the ribosomal protein (Rp49) primers were used; Rp49 Fwd: 5'-TGTTGTGTCCTTCCAGCTTCAA-3'; and Rp49 Rev: 5'-ACTGATATCCATCCATCCAGATAATG-3'.

### 4.3. 5' Rapid Amplification of cDNA Ends

A clone of the 5' region of *Snama* was obtained from total RNA by using the GeneRacer™ kit (Invitrogen) following the manufacturer’s protocol with slight modifications. Approximately 5 μg total RNA were treated with calf intestinal phosphatase (CIP) to remove 5' terminal phosphates. The “cap” from mRNA was then removed by treatment of the total RNA with tobacco acid pyrophosphatase (TAP), thereby exposing the 5' terminal phosphates. This facilitated the ligation of the RNA oligonucleotide (5'-CGACUGGAGCACGAGGACACUGACAUGGACUGAAGGAGUAGAAA-3') to the TAP-treated mRNA using T4 RNA ligase. This was then followed by RT-PCR. All steps were carried out as described in the GeneRacer™ kit using the following oligonucleotide primers: GeneRacer™ 5' (GR5'): 5'-CGACTGGAGCACGAGGACACTGA-3'; GeneRacer™ 5' nested (GR5nested): 5'-GGACACTGACATGGACTGAAGGAGTA-3'; RING finger (RF) reverse: 5'-CGAACAAAGCTTCTCCTTGCAATCG-3'; RF tail: 5'-AGTGAGTGCCCCGATTGCAAGGAG-3'; and RF tail internal: 5'-CCGCAGCAGGGTATCATGACAGCAT-3'. The ImProm-II™ or SuperScript™ II reverse transcription systems were used to generate cDNA from *Drosophila* mRNA and from the HeLa control mRNA. The RF tail primer was used as a gene-specific primer during the reverse transcription reaction, while an oligo(dT) primer was used for the HeLa control mRNA. Extension temperatures for these reactions were 55 and 42 °C, respectively.

### 4.4. Preparation of Reporter Constructs and Assays

A series of three 5' truncated fragments were created by PCR amplification from genomic DNA isolated from wild-type Drosophila melanogaster using the following primers that are based on the region stretching up to 672 bases upstream of the transcription start site (underlined in the reverse primer): Promoter 1 (Fwd): 5'-GACTAAATTAGATCTGCATCGA-3'; Promoter 2 (Fwd): 5'-GCAAAGATCTAAAAGGCGGTG-3'; Promoter 3 (Fwd): 5'-GCCTCCTTAAGATCTATTCG-3'; reverse primer: 5'-GAACGTGACGCCAAAGCCC-3'. The PCR products were cloned into the pGL3 basic vector, and recombinant constructs were used to transiently transfect Cos7 cells using the SuperFect Transfection Reagent (Qiagen, Limburg, The Netherlands) according to the manufacturer’s instructions. We used the Dual luciferase system (Promega, Madison, VA, USA) to measure the firefly and Renilla luciferase activities sequentially. Cells were cultured until they reached a density of 1 × 10^5^ cells/mL (in 5 mL), after which, they were transfected with 5 μg of DNA. The ratio of the experimental vector to the control vector was 1:20.

Assays were carried out in triplicate, and the data were used to plot the graph using relative ratios as follows:

(1) $$X=\frac{control\left(firefly\;luminescence\right)}{control\left(renilla\;luminescence\right)}$$

(2) $$Y=\frac{experimental\left(firefly\;luciferase\right)}{experimental(renilla\;luciferase)}$$

(3) $$Relative\;ratio=\frac{Y}{X}$$

Generally, a total of 5 µg of DNA (recombinant pGL3 basic vector and pRL-TK 1:20) was added to growth medium lacking both antibiotic and serum to a volume of 150 µL followed by 30 µL of the SuperFect Transfection Reagent. After incubation for 10 min at 25 °C, 1 mL of medium with fetal bovine serum (FBS) and penicillin-streptomycin (PenStrep) were added. This mixture was added to cells that had been washed in 1× PBS and incubated for 3 h at 37 °C in 5% CO_2_. Fresh growth medium was then added, and the cells were incubated further under normal growth conditions (37 °C and 5% CO_2_) for 48 h. The luciferase assay was performed by using the Dual-Glo Luciferase Assay System (Promega) in 96-well luminometer plates following the manufacturer’s instructions. The luciferase and Renilla luminescence activities were then measured in the GloMax^®^ 96 Microplate Luminometer.

### 4.5. Fractionation of Cell Extracts

Nuclear extracts from 0–6 h embryos were prepared according to the method of Tie *et al.* [54]. The extract remaining after removal of the nuclei was fractionated into post-nuclear and cytosolic fractions by differential centrifugation at 4 °C at 3000× *g* and at 18,000× *g* for 10 and 30 min, respectively. The post-nuclear extract contains small organelles and macromolecular structures, such as ribosomes, the Golgi apparatus and the endoplasmic reticulum and sediments at 3000–18,000× *g*, while the remaining cellular content in the post 18,000× *g* fraction constitutes the cytosolic fraction.

### 4.6. Modification of RS Domain Proteins: Acetylation and Phosphorylation and Mg^+2^-Precipitation

Acetylation was performed as described by [6], which was based on [55]. Phosphorylation and subsequent Mg^2+^-precipitation were performed as described by [40]. The basis for this selective precipitation technique is phosphorylation of serine-rich tracts constituting the RS-domain, with Mg^2+^ then forming intra- and-inter ionic cross-links between phosphoserines and resultant precipitation.

### 4.7. Electromobility Shift Assays and Streptavidin Chromatography

Labelled Promoter Fragments 1, 2 and 3 were generated by PCR using biotin-tagged primers. A nuclear extract obtained from 0–6 h embryos was added to PCR products to enable protein-DNA interactions according to the Lightshift EMSA kit (Thermo Scientific Catalogue#20148). The resulting protein-DNA complexes were added to streptavidin agarose beads that were pre-washed in the binding buffer provided in the kit followed by incubation for 30 min. The beads were pelleted by centrifugation and washed in the washing buffer also provided in the kit. Typically, bound proteins were separated on a 12% SDS-PAGE gel.

### 4.8. Preparation of Antibodies against SNAMA-DCM

Recombinant proteins were purified from extracts of *E. coli* BL21(DE3)pLysS that was induced to express either the 26-kDa GST and HIS-tag proteins or the tags fused with SNAMA-DCM. Polyclonal antibodies (immunoglobulin Y (IgY)) were raised in chickens against a recombinant fusion protein containing the first 260 amino-terminal residues comprising the DWNN catalytic module (DCM) fused to a glutathione *S*-transferase (GST)-tag and flanked by two His-tag peptides. The recombinant protein was confirmed by peptide sequencing using MALDI-TOF to be SNAMA DCM. To purify the anti-SNAMA DCM antibodies, two affinity columns were created by reductive amidation, employing sodium cyanoborohydride (Sigma-Aldrich) as the reducing agent and aldehyde-agarose (Sigma-Aldrich) as a support matrix: one with the SNAMA DCM fusion protein immobilized on the matrix and the other with the recombinant GST/HIS protein. Anti-SNAMA DCM antibodies were selected from the post-immune IgY on the first column and the contaminating antibodies on the second column to exclude anti-GST and anti-His-tag antibodies. The pre-immune IgY was also pre-adsorbed on the second column for use as a negative control.

### 4.9. Co-Immunoprecipitation Assays

Co-immunoprecipitation assays were performed according to [56], with some modifications. Duplicate experiments, containing one milligram of crude protein extracts from either 0–6 h embryos, mixed stages of larvae or male flies were conducted. In each, 15 µg of anti-SNAMA DCM antibody or the same amount of pre-adsorbed pre-immune serum were added. Immune-complexes were captured on 20 µL of chicken IgY precipitating resin (GenScript, Piscataway, NJ, USA) washed several times, solubilized in sample buffer and separated by SDS-PAGE. Gels were then scanned by a densitometer (Bio-Rad, Hercules, CA, USA, Model GS800). Band identification and analysis were performed by using the accompanying Quantity One^®^ software package.

### 4.10. Preparation of Proteins from Gels and Identification via Tandem Mass Spectrometry

Coomassie-stained samples were stored in 5% acetic acid, whereas silver-stained samples were first destained according to [57], except for minor changes. Briefly, the bands were destained with 1 mL destaining solution (500 µL 30 mM potassium ferricyanide and 500 µL 100 mM sodium thiosulfate) for 2–2.5 h. They were then washed twice with 1 mL distilled water for 10 min each time. Having transferred them into Eppendorf tubes, 0.5 mL 200 mM ammonium bicarbonate was added to each sample followed by storage at 4 °C.

Selected protein bands were in-gel trypsin digested as described in [58]. In short, the bands were destained using 50 mM NH_4_HCO_3_/50% methanol followed by in-gel protein reduction (50 mM DTT in 25 mM NH_4_HCO_3_) and alkylation (55 mM iodoacetamide in 25 mM NH_4_HCO_3_). The proteins were then digested overnight at 37 °C using 5–50 µL, 10 ng/µL trypsin, depending on the size of the gel piece. Digests were resuspended in 35 µL, 2% acetonitrile/0.2% formic acid and analyzed using a Dionex Ultimate 3000 Rapid Separation Liquid Chromatography (RSLC) system coupled to a QSTAR ELITE mass spectrometer. Peptides were first de-salted on an Acclaim PepMap C18 trap column (75 μm × 2 cm) for 8 min at 5 μL/min using 2% acetonitrile/0.2% formic acid, then separated on an Acclaim PepMap C18 RSLC column (75 μm × 15 cm, 2-µm particle size). Peptides were eluted using a flow-rate of 500 μL/min with a gradient: 4%–60% B in 30 min (A: 0.1% formic acid; B: 80% acetonitrile/0.1% formic acid). Nano-spray was achieved using a MicroIonSpray head assembled with a New Objective, PicoTip emitter. An electrospray voltage of 2.0–2.8 kV was applied to the emitter. The QSTAR ELITE mass spectrometer was operated in information-dependent acquisition using an exit factor of 7.0 and a maximum accumulation time of 2.5 s. MS scans were acquired from *m*/*z* 400–1500, and the three most intense ions were automatically fragmented in Q2 collision cells using nitrogen as the collision gas. Collision energies were chosen automatically as a function of *m*/*z* and charge.

ProteinPilot™ v4.0.8085 using the Paragon search engine (AB SCIEX) was used for comparison of the MS/MS spectra against *Drosophila melanogaster* protein sequences in the Microsoft system database (msdb). Generally, proteins with a threshold close to 90% confidence and higher were reported.

### 4.11. Western Blot Analysis

Proteins were separated by SDS-PAGE and transferred to a Hybond-P PVDF membrane (Amersham Bioscience, Sunnyvale, CA, USA) in a semi-dry blotter. Non-specific binding was prevented by blocking with SuperBlock™ Dry Blend Blocking Buffer (Pierce, Rockford, IL, USA). The blot was probed with a 1:5000 dilution of chicken anti-SNAMA DCM antibody. A 1:10,000 dilution of anti-chicken IgY peroxidase conjugate was used as a secondary antibody. The signal generated by the SuperSignal^®^ Chemiluminescent substrate kit (Pierce) was visualized by exposing the blot to X-ray film.

### 4.12. Immunocytochemical Staining of Whole Mount Embryos and Ovaries

Embryos were collected over apple juice agar and were prepared for immunolabeling as described by White [59], except that they were devitellinized with ethanol. SNAMA was detected with 1:500 dilution of rabbit anti-SNAMA antibody followed by incubation in 1/100 dilution of HRP-labelled anti-rabbit antibody (Sigma Aldrich). The peroxidase reaction was visualized with diaminobenzidine tetrahydrochloride (DAB) enhanced with a solution of 5% NiCl_2_·6H_2_O and 5% CoCl_2_·H_2_O in ddH_2_O. Embryos were then washed three times in PBTX (PBS, 0.1% BSA, 0.1% Triton X-100), three times in PT (PBS, 0.1% Tween 20) and passed through 30%, 50% and then stored in 80% glycerol in PBS.

Ovaries were dissected, prepared for immunolabeling and stained with the above antibodies as described by [60]. Egg chambers were dissected before the staining procedure.

### 4.13. Immunofluorescence and Confocal Microscopy

Embryos were fixed, stored in methanol at −20 °C and stained as described previously by White [59]. Ovaries were prepared as described by Verheyen and Cooley [60]. A 1:500 dilution of the polyclonal anti-SNAMA antibody was used, followed by 1:500 dilution of anti-rabbit antibody conjugated with FITC (green) (Sigma-Aldrich) as the secondary antibody. The embryos were mounted in glycerol with *p*-phenylenediamine (Sigma-Aldrich) as an anti-fade agent. Nuclei-containing samples were treated with RNase A (Biobasic Inc., Markham, ON, Canada) and counterstained with propidium iodide, which is responsible for the red color. Anti-fade was not applied to ovary samples. Confocal images were captured on a Zeiss 510 META Confocal Laser Scanning Microscope.

### 4.14. Bioinformatics Analysis

The Patch program was used to search the Transfac database for insect transcription factors. Potential p53 binding sites were detected by using the special algorithm designed for p53 [20].

## 5. Conclusions

We have shown that *Snama* is expressed as two developmentally regulated transcripts that are produced by alternative splicing. The promoter region was defined, and putative transcription factor binding sites were predicted. These results are consistent with the general understanding that RBBP6 family members play key roles in cell proliferation and in pre-mRNA processing. Furthermore, these data suggest that SNAMA may be associated with embryonic patterning, especially of the anteroposterior axis. These functional features reinforce the current view that members of the RBBP6 family are important and play crucial roles that have implications for diseases such as cancer.

## References

[1] Mbita Z., Meyer M., Skepu A., Hosie M., Rees J., Dlamini Z. (2012). De-regulation of the RBBP6 isoform 3/DWNN in human cancers. Mol. Cell. Biochem..

[2] Yoshitake Y., Nakatsura T., Monji M., Senju S., Matsuyoshi H., Tsukamoto H., Hosaka S., Komori H., Fukuma D., Ikuta Y. (2004). Proliferation potential-related protein, an ideal esophageal cancer antigen for immunotherapy, identified using complimentary DNA microarray analysis. Clin. Cancer Res..

[3] Chen J., Tang H., Wu Z., Zhou C., Jiang T., Xue Y., Huang G., Yan D., Peng Z. (2013). Overexpression of RBBP6, alone or combined with mutant TP53, is predictive of poor prognosis in colon cancer. PLoS ONE.

[4] Motadi L.R., Bhoola K.D., Dlamini Z. (2011). Expression and function of retinoblastoma binding protein 6 (RBBP6) in human lung cancer. Immunobiology.

[5] Harutyunyan A.S., Giambruno R., Krendl C., Stukalov A., Klampfl T., Berg T., Milosevic J.D., Chen D., Gisslinger B., Gisslinger H. (2013). Germline RBBP6 mutations in myeloproliferative neoplasms. Blood.

[6] Simons A., Melamed-Bessudo C., Wolkowicz R., Sperling J., Sperling R., Eisenbach L., Rotter V. (1997). PACT: Cloning and characterization of a cellular p53 binding protein that interacts with Rb. Oncogene.

[7] Witte M.M., Scott R.E. (1997). The proliferation potential protein-related (*P2P-R*) gene with domains encoding heterogeneous nuclear ribonucleoprotein association and Rb1 binding shows repressed expression during terminal differentiation. Proc. Natl. Acad. Sci. USA.

[8] Mather A., Rakghotho M., Ntwasa M. (2005). SNAMA, a novel protein with a DWNN domain and a RING finger-like motif: A possible role in apoptosis. Biochim. Biophys. Acta.

[9] Pretorius A., Kaur M., Wamalwa M., February M.F., Essack M., Bajic V.B., Rees D.J.G. (2013). Functional analysis and characterization of the human RBBP6 promoters based on a combination of molecular biology and *in silico* approaches provide additional evidence for RBBP6 role in apoptosis. J. Biosci..

[10] Ntwasa M. (2008). The retinoblastoma binding protein 6 is a potential target for therapeutic drugs. Biotechnol. Mol. Biol. Rev..

[11] Chibi M., Meyer M., Skepu A., Rees D.J.G., Moolman-Smook J.C., Pugh D.J.R. (2008). RBBP6 Interacts with multifunctional protein YB-1 through its RING finger domain, leading to ubiquitination and proteosomal degradation of YB-1. J. Mol. Biol..

[12] Pugh D., Ab E., Faro A., Lutya P., Hoffmann E., Rees D.J. (2006). DWNN, a novel ubiquitin-like domain, implicates RBBP6 in mRNA processing and ubiquitin-like pathways. BMC Struct. Biol..

[13] Di Giammartino D.C., Li W., Ogami K., Yashinskie J.J., Hoque M., Tian B., Manley J.L. (2014). RBBP6 isoforms regulate the human polyadenylation machinery and modulate expression of mRNAs with AU-rich 3' UTRs. Genes Dev..

[14] Jones C., Reifegerste R., Moses K. (2006). Characterization of *Drosophila mini-me*, a gene required for cell proliferation and survival. Genetics.

[15] Li L., Deng B., Xing G., Teng Y., Tian C., Cheng X., Yin X., Yang J., Gao X., Zhu Y. (2007). PACT is a negative regulator of p53 and essential for cell growth and embryonic development. Proc. Natl. Acad. Sci. USA.

[16] Lane D.P., Verma C. (2012). Mdm2 in Evolution. Genes Cancer.

[17] Huang P., Ma X., Zhao Y., Miao L. (2013). The *C. elegans* homolog of RBBP6 (RBPL-1) regulates fertility through controlling cell proliferation in the germline and nutrient synthesis in the intestine. PLoS ONE.

[18] Gao S., Scott R.E. (2002). P2P-R protein overexpression restricts mitotic progression at prometaphase and promotes mitotic apoptosis. J. Cell. Physiol..

[19] Miotto B., Chibi M., Xie P., Koundrioukoff S., Moolman-Smook H., Pugh D., Debatisse M., He F., Zhang L., Defossez P.A. (2014). The RBBP6/ZBTB38/MCM10 axis regulates DNA replication and common fragile site stability. Cell Rep..

[20] Hoh J., Jin S., Parrado T., Edington J., Levine A.J., Ott J. (2002). The p53MH algorithm and its application in detecting p53-responsive genes. Proc. Natl. Acad. Sci. USA.

[21] Pignoni F., Steingrimsson E., Lengyel J.A. (1992). bicoid and the terminal system activate tailless expression in the early Drosophila embryo. Development.

[22] Mlodzik M., Gehring W.J. (1987). Hierarchy of the genetic interactions that specify the anteroposterior segmentation pattern of the Drosophila embryo as monitored by caudal protein expression. Development.

[23] Han K., Manley J.L. (1993). Functional domains of the Drosophila Engrailed protein. EMBO J..

[24] Brunner E., Peter O., Schweizer L., Basler K. (1997). Pangolin encodes a Lef-1 homologue that acts downstream of Armadillo to transduce the Wingless signal in *Drosophila*. Nature.

[25] England B.P., Admon A., Tjian R. (1992). Cloning of Drosophila transcription factor Adf-1 reveals homology to Myb oncoproteins. Proc. Natl. Acad. Sci. USA.

[26] Luo S.D., Shi G.W., Baker B.S. (2011). Direct targets of the D. melanogaster DSXF protein and the evolution of sexual development. Development.

[27] Hsu T., Gogos J., Kirsh S., Kafatos F. (1992). Multiple zinc finger forms resulting from developmentally regulated alternative splicing of a transcription factor gene. Science.

[28] Hsu T., Bagni C., Sutherland J.D., Kafatos F.C. (1996). The transcriptional factor CF2 is a mediator of EGF-R-activated dorsoventral patterning in *Drosophila* oogenesis. Genes Dev..

[29] Matsukage A., Hirose F., Yoo M.-A., Yamaguchi M. (2008). The DRE/DREF transcriptional regulatory system: A master key for cell proliferation. Biochim. Biophys. Acta Gene Regul. Mech..

[30] Baylies M.K., Bate M. (1996). Twist: A myogenic switch in Drosophila. Science.

[31] Yang J., Mani S.A., Donaher J.L., Ramaswamy S., Itzykson R.A., Come C., Savagner P., Gitelman I., Richardson A., Weinberg R.A. (2004). Twist, a master regulator of morphogenesis, plays an essential role in tumor metastasis. Cell.

[32] Tanaka K.K.K., Bryantsev A.L., Cripps R.M. (2008). Myocyte enhancer factor 2 and chorion factor 2 collaborate in activation of the myogenic program in *Drosophila*. Mol. Cell. Biol..

[33] Cripps R.M., Black B.L., Zhao B., Lien C.-L., Schulz R.A., Olson E.N. (1998). The myogenic regulatory gene *Mef2* is a direct target for transcriptional activation by Twist during Drosophila myogenesis. Genes Dev..

[34] Jiang N., Emberly E., Cuvier O., Hart C.M. (2009). Genome-wide mapping of boundary element-associated factor (BEAF) binding sites in *Drosophila melanogaster* links BEAF to transcription. Mol. Cell. Biol..

[35] Gilbert M.K., Tan Y.Y., Hart C.M. (2006). The Drosophila boundary element-associated factors BEAF-32A and BEAF-32B affect chromatin structure. Genetics.

[36] Raff J.W., Kellum R., Alberts B. (1994). The *Drosophila* GAGA transcription factor is associated with specific regions of heterochromatin throughout the cell cycle. EMBO J..

[37] Morris D.R., Geballe A.P. (2000). Upstream open reading frames as regulators of mRNA translation. Mol. Cell. Biol..

[38] Wilkie G.S., Dickson K.S., Gray N.K. (2003). Regulation of mRNA translation by 5'- and 3'-UTR-binding factors. Trends Biochem. Sci..

[39] Coll O., Villalba A., Bussotti G., Notredame C., Gebauer F. (2010). A novel, noncanonical mechanism of cytoplasmic polyadenylation operates in Drosophila embryogenesis. Genes Dev..

[40] Blencowe B.J., Issner R., Kim J., McCaw P., Sharp P.A. (1995). New proteins related to the Ser-Arg family of splicing factors. RNA.

[41] Hart C.M., Cuvier O., Laemmli U.K. (1999). Evidence for an antagonistic relationship between the boundary element-associated factor BEAF and the transcription factor DREF. Chromosoma.

[42] Hirose F., Ohshima N., Shiraki M., Inoue Y.H., Taguchi O., Nishi Y., Matsukage A., Yamaguchi M. (2001). Ectopic expression of DREF induces DNA synthesis, apoptosis, and unusual morphogenesis in the *Drosophila* eye imaginal disc: Possible interaction with polycomb and trithorax group proteins. Mol. Cell. Biol..

[43] Meinhart A., Cramer P. (2004). Recognition of RNA polymerase II carboxy-terminal domain by 3(prime)-RNA-processing factors. Nature.

[44] Pauli D., Tonka C.H., Tissieres A., Arrigo A.P. (1990). Tissue-specific expression of the heat shock protein HSP27 during Drosophila melanogaster development. J. Cell Biol..

[45] Michaud S., Lavoie S., Guimond M.-O., Tanguay R.M. (2008). The nuclear localization of Drosophila Hsp27 is dependent on a monopartite arginine-rich NLS and is uncoupled from its association to nuclear speckles. Biochim. Biophys. Acta Mol. Cell Res..

[46] Foe V.E., Odell G.M., Edgar B.A., Bate M., Arias A.M. (1993). Mitosis and morphogenesis in the *Drosophila* embryo: Point and counterpoin. The Development of Drosophila Melanogaster.

[47] De Oca Luna R.M., Wagner D.S., Lozano G. (1995). Rescue of early embryonic lethality in mdm2-deficient mice by deletion of p53. Nature.

[48] Mach J.M., Lehmann R. (1997). An Egalitarian-BicaudalD complex is essential for oocyte specification and axis determination in Drosophila. Genes Dev..

[49] Bullock S.L., Ish-Horowicz D. (2001). Conserved signals and machinery for RNA transport in *Drosophila* oogenesis and embryogenesis. Nature.

[50] Clark K.A., McKearin D.M. (1996). The Drosophila stonewall gene encodes a putative transcription factor essential for germ cell development. Development.

[51] Maines J.Z., Park J.K., Williams M., McKearin D.M. (2007). Stonewalling Drosophila stem cell differentiation by epigenetic controls. Development.

[52] Manoli D.S., Meissner G.W., Baker B.S. (2006). Blueprints for behavior: Genetic specification of neural circuitry for innate behaviors. Trends Neurosci..

[53] Crozatier M., Kongsuwan K., Ferrer P., Merriam J.R., Lengyel J.A., Vincent A. (1992). Single amino acid exchanges in separate domains of the Drosophila serendipity delta zinc finger protein cause embryonic and sex biased lethality. Genetics.

[54] Tie F., Furuyama T., Prasad-Sinha J., Jane E., Harte P.J. (2001). The Drosophila Polycomb Group proteins ESC and E(Z) are present in a complex containing the histone-binding protein p55 and the histone deacetylase RPD3. Development.

[55] Bayer E.A., Ehrlich-Rogozinski S., Wilchek M. (1996). Sodium dodecyl sulfate-polyacrylamide gel electrophoretic method for assessing the quaternary state and comparative thermostability of avidin and streptavidin. Electrophoresis.

[56] Xu Z., Gong Q., Xia B., Groves B., Zimmermann M., Mugler C., Mu D., Matsumoto B., Seaman M., Ma D. (2009). A role of histone H3 lysine 4 methyltransferase components in endosomal trafficking. J. Cell Biol..

[57] Gharahdaghi F., Weinberg C.R., Meagher D.A., Imai B.S., Mische S.M. (1999). Mass spectrometric identification of proteins from silver-stained polyacrylamide gel: A method for the removal of silver ions to enhance sensitivity. Electrophoresis.

[58] Shevchenko A., Tomas H., Havlis J., Olsen J.V., Mann M. (2007). In-gel digestion for mass spectrometric characterization of proteins and proteomes. Nat. Protoc..

[59] White R.A.H., Roberts D.B. (1998). Immunolabelling of drosophila. Drosophila: A Practical Approach.

[60] Verheyen E., Cooley L., Goldstein L.S.B., Fyrberg E.A. (1994). Looking at Oogenesis. Drosophila Melanogaster: Practical Uses in Cell and Molecular Biology.

